# Comparison of mucosal microbiota populations across the gastrointestinal tract of healthy dogs

**DOI:** 10.1186/s42523-024-00368-7

**Published:** 2025-01-06

**Authors:** Ching-Yen Lin, Tzu-Wen L. Cross, Kelly S. Swanson

**Affiliations:** 1 Division of Nutritional Sciences, University of Illinois Urbana-Champaign, Urbana, IL 61801 USA; 2 Department of Animal Sciences, University of Illinois Urbana-Champaign, Urbana, IL 61801 USA

**Keywords:** Age, Canine, Diet, Gastrointestinal, Mucosal microbiota

## Abstract

The gastrointestinal (GI) microbiota plays a crucial role in host health and disease in dogs, but the knowledge regarding the mucosal associated microbiota along the GI tract is limited in dogs. Therefore, the objective of this study was to characterize the phylogeny and predicted functional capacity of microbiota residing on the gut mucosa across five GI regions of healthy young adult and geriatric dogs fed different diets. Twelve weanling (8 weeks old) and 12 senior (11.1 years old) beagles were randomly assigned to be fed an animal product-based diet or plant product-based diet for 12 months. At that time, mucosal samples from the stomach, duodenum, jejunum, ileum, and mid-colon were collected. DNA was extracted and the hypervariable region 4 (V4) of the 16S rRNA gene was amplified to assess microbiota using Illumina MiSeq sequencing. Data were analyzed using QIIME 1.9.1. and Statistical Analyses of Metagenomic Profiles software 2.1.3. Gene predictions were made using Phylogenetic Investigation of Communities by Reconstruction of Unobserved States. Taxonomic assessment revealed a greater (*p* < 0.05) species richness in the mid-colon compared with other segments. Principal coordinates analysis of weighted UniFrac distances demonstrated distinct clusters of stomach, ileum, and mid-colon samples, indicating the presence of unique microbial communities in these regions. The predominant phyla in all five segments were Proteobacteria, Firmicutes, Bacteroidetes, Actinobacteria and Fusobacteria, but their relative abundances varied between segments. Proteobacteria had a decreasing relative abundance from the stomach to mid-colon (*q* < 0.05). The ileum had the highest while the stomach had the lowest relative abundance of Firmicutes (*q* < 0.05). The duodenum had a higher abundance of Bacteroidetes than the stomach and ileum (*q* < 0.05). The mid-colon had a higher Fusobacteria relative abundance than other regions (*q* < 0.05). The predicted functional capacities of the microbiota in the stomach differed from those in the other segments. Age and diet of dog did not significantly impact the taxonomy or predicted functional capacities of the mucosal microbiota. In conclusion, our findings demonstrate distinct characteristics of the mucosal microbiota across various segments of canine GI tract.

**Supplementary Information:**

The online version contains supplementary material available at 10.1186/s42523-024-00368-7.

## Background

The mammalian gastrointestinal (GI) tract is home to microbiota encompassing microorganisms such as bacteria, viruses, archaea, and eukaryotes. This complex microbial community, collectively known as the GI microbiota, plays a vital role in maintaining host health. It functions as both an immune organ, defending against intestinal pathogens and regulating the immune system, and a metabolic organ, providing energy sources like short-chain fatty acids to intestinal epithelial cells. Alterations in the composition and function of the gut microbiota have been associated with various disorders.

Studies have shown that dogs with GI diseases, such as chronic enteropathies or hemorrhagic gastroenteritis, exhibit distinct alterations in their fecal microbial composition compared with healthy dogs [1, 2]. Furthermore, changes in the GI microbiota have also been observed in dogs with obesity and diabetes [3–5]. Thus, the gut microbiota holds promise as biomarkers for future diagnostic and monitoring purposes associated with diseases. However, given that most studies have reported microbiota results from fecal samples, there’s a notable gap in our knowledge about the microbial populations residing within the GI tract of dogs.

The mucosal microbiota, which adheres to the intestinal mucosa, offers unique insights into the intimate interaction between microbes and the host, potentially influencing local and systemic physiological processes. Two studies have demonstrated the distinct variation of microbial compositions and characterized the metabolome of intestinal contents across the GI tract (duodenum, jejunum, ileum, colon, and rectum) of healthy adult dogs [6, 7]. These studies revealed a gradual shift in the abundance of microbial taxa along the GI tract, with some experiencing a sudden decrease towards the end of the small intestine, reflecting the distinct microenvironment and physiological differences of each GI tract segment. Although variation of microbial composition was observed across the canine GI tract, the majority of the bacterial sequences are classified into one of five phyla: Proteobacteria, Firmicutes, Fusobacteria, Bacteroidetes, and Actinobacteria.

While some studies have investigated the variation of microbial taxonomy and metabolomes of intestinal contents across the GI tract in healthy adult dogs, the influence of factors that significantly impact the gut microbiota such as diet, age, and sex have not been reported on the GI microbiota of dogs. Diets play a significant role in shaping the composition of the GI microbiota. Several studies have reported changes in fecal microbial composition when dogs consumed diets vary in macronutrient composition, degrees of diet processing, or the inclusion of specific ingredients such as dietary fibers or prebiotics [8–13]. Additionally, age has been identified as another key factor influencing the fecal microbiota, with declining microbial diversity accompanying increasing age [14]. The abundance of specific bacterial taxa within the fecal microbiota, such as *Lactobacillus* and *Fusobacterium perfoetens*, have also been reported to change with age in healthy dogs [15, 16]. However, these studies primarily assessed the GI microbiota using feces as proxy, and limited research exists on the effects of age or diet on the canine mucosal microbiota.

Given the lack of published literature profiling the mucosal microbiota in healthy dogs, the primary objective of this study was to comprehensively characterize the microbial composition and predict the functional capacity of mucosal bacteria across multiple GI segments, including the stomach, duodenum, jejunum, ileum, and colon in dogs. The secondary objective was to investigate the impact of diet (animal protein-based vs. plant protein-based) and age on the mucosal microbiota. By investigating the mucosal microbiota in different GI segments and considering the influence of age, diet, and health status, we seek to enhance our understanding of the canine GI ecosystem and its potential associations with dietary influences and age-related changes in dogs.

## Materials and methods

### Animal and diets

All animal care and handling are detailed in Swanson et al. [17] and all the experimental procedures were approved by the University of Illinois Institutional Animal Care and Use Committee prior to the initiation of the study (Protocol No. 02056). Briefly, 12 senior (average age = 11.1 ± 0.6 year; 6 males and 6 females) and 12 weanling (8 wk old; 6 males and 6 females) beagles (Marshall Farms USA, Inc., North Rose, NY) were used in this study. Dogs were housed individually in kennels (1.1 × 0.9 m) in temperature-controlled rooms with a 12-h light:12-h dark cycle at the Edward R. Madigan Laboratory on the University of Illinois campus. All dogs were randomly assigned and feed to one of two extruded kibble diets (animal product-based diet or plant product-based diet) and fed for 12 mo (Table 1). The animal product-based diet was mainly composed of highly digestible animal-derived ingredients, while the plant product-based diet was primarily composed of moderately digestible plant-derived ingredients (Table 1). Both diets were formulated to meet al.l the nutrient recommendations for canine growth and reproduction according to Association of American Feed Control Officials (AAFCO, 2003). Young dogs were fed *ad libitum* throughout the experiment, while senior dogs were fed to maintain body weight using the weight at the beginning of the study as the target. Dogs were housed individually in environmentally controlled rooms with a 12-h light: 12-h dark cycle at the Edward R. Madigan Laboratory on the University of Illinois at Urbana-Champaign campus.

**Table 1:** Ingredient and chemical composition of the animal product-based (APB) and plant product-based (PPB) diets fed to dogs

Ingredient	APB^1^	PPB^2^
	--- %, as-is ---	
Corn	-	45.00
Brewer’s rice	44.23	-
Chicken by-product meal	32.91	-
Soybean meal	-	19.96
Poultry fat	14.99	3.97
Wheat middlings	-	13.20
Meat and bone meal	-	10.00
Beet pulp	4.00	4.00
Dehydrated egg	2.20	2.20
Sodium chloride	0.65	0.65
Potassium chloride	0.65	0.65
Vitamin premix^3^	0.13	0.13
Mineral premix^3^	0.12	0.12
**Analyzed composition**		
Dry matter	93.8	94.3
	--- % of DM ---	
Organic matter	92.8	92.3
Ash	7.2	7.7
Crude protein	28.0	25.5
Acid-hydrolyzed fat	22.6	11.2
Total dietary fiber	4.8	15.2
ME, kcal/g^4^	4.2	3.3

^1^ Provided per kg of APB diet: choline, 2654 mg; retinyl acetate, 15.2 KIU; cholecalciferol, 0.9 KIU; alpha-tocopherol, 62.5 IU; menadione sodium bisulfite complex (source of vitamin K), 0.6 mg; thiamin, 13.1 mg; riboflavin, 14.0 mg; pantothenic acid, 25.3 mg; niacin, 70.0 mg; pyridoxine, 13.56 mg; biotin, 0.11 mg; folic acid, 949 µg; vitamin B-12, 129 µg; manganese (as MnSO_4_), 19.6 mg; iron (as FeSO_4_), 253.9 mg; copper (as CuSO_4_), 17.8 mg; cobalt (as CoSO_4_), 2.4 mg; zinc (as ZnSO_4_), 166.9 mg; iodine (as KI), 6.3 mg; and selenium (as Na_2_SeO_3_), 0.32 mg

^2^ Provided per kg of PPB diet: choline, 2457 mg; retinyl acetate, 16.3 KIU; cholecalciferol, 0.9 KIU; alpha-tocopherol, 74.1 IU; menadione sodium bisulfite complex (source of vitamin K), 1.2 mg; thiamin, 14.4 mg; riboflavin, 11.5 mg; pantothenic acid, 23.9 mg; niacin, 79.3 mg; pyridoxine, 15.8 mg; biotin, 0.24 mg; folic acid, 1024 µg; vitamin B-12, 33.3 µg; manganese (as MnSO_4_), 24.0 mg; iron (as FeSO_4_), 214.6 mg; copper (as CuSO_4_), 23.1 mg; cobalt (as CoSO_4_), 2.4 mg; zinc (as ZnSO_4_), 144.3 mg; iodine (as KI), 24.0 mg; selenium (as Na_2_SeO_3_), 0.27 mg

^3^ Trouw Nutrition USA, LLC, Highland, IL

^4^ Metabolizable energy (ME, kcal/kg) = (3.5 kcal/g × crude protein %) + (8.5 kcal/g × acid-hydrolyzed fat %) + (3.5 kcal/g × nitrogen-free extract %); nitrogen-free extract (%) = 100% - (crude protein % + acid-hydrolyzed fat % + ash % + total dietary fiber %)

### Sample collection

After 12 mo on experiment, dogs were fasted for 12 h and euthanized using sodium pentobarbital (130 mg/kg body weight; Euthasol^®^, Virbac Corp., Fort Worth, TX). Intestinal samples were collected from 5 regions: the stomach, the duodenum (10 cm distal to the pyloric sphincter), jejunum (10 cm distal to ligament of treitz), ileum (10 cm proximal to the ileocecal junction) and colon (midpoint). All samples were quickly frozen in liquid nitrogen and then stored at -80℃ until analyses. All samples were collected within 20 min of the time of death.

### DNA extraction, amplification, and sequencing

Mucosal samples were scraped from the tissue using microscope slide cover slips. Total DNA from mucosal samples was extracted using Mo-Bio PowerSoil Kits (MO BIO laboratories, Inc., Carlsbad, CA), followed by quantification of extracted DNA using a Qubit^®^ 3.0 Fluorometer (Life Technologies, Grand Island, NY). Bacterial 16S rRNA gene amplicons of 252 bp from the V4 region were generated using a Fluidigm Access Array (Fluidigm Corporation, South San Francisco, CA) with Roche High Fidelity Fast Start Kit (Roche, Indianapolis, IN). The primers 515 F (5’-GTGCCAGCMGCCGCGGTAA-3’) and 806R (5’-GGACTACHVGGGTWTCTAAT-3’) that target the V4 region were used for amplification (primers synthesized by IDT Corp., Coralville, IA) [18]. Quality of the amplicons was accessed using a Fragment Analyzer (Advanced Analytics, Ames, IA) followed by amplicon size selection using electrophoresis and Qiagen gel purification kit (Qiagen, Valencia, CA). The appropriate profile and average size of purified amplicons were then confirmed using an Bioanalyzer (Agilent Technologies, Santa Clara, CA). Amplicons were sequenced using Illumina sequencing on a MiSeq using v3 reagents (Illumina Inc., San Diego, CA) at the Roy J. Carver Biotechnology Center at the University of Illinois.

### Bioinformatics and statistical analyses

Forward reads were trimmed using the FASTX-Toolkit (version 0.0.14), and QIIME 1.9.1 [19] was used to process the resulting sequence data. High-quality (quality value ≥ 20) sequence data derived from the sequencing process were demultiplexed. Sequences then were clustered into operational taxonomic units (OTU) using UCLUST [20] through an open-reference OTU picking strategy against the Greengenes 13_8 reference database [21] with a 97% similarity threshold. OTU that had less than 0.01% of the total observation were discarded. Taxonomic identity to each OTU was then assigned using UCLUST. A total of 6,269,120 16S rRNA-based amplicon sequences were obtained, with an average of 728,96 reads per sample. An even sampling depth (sequences per sample) of 2,229 sequences per sample was used for assessing alpha- and beta-diversity measures. Alpha diversity of the microbiota was estimated using phylogenetic diversity (PD) whole tree, Chao1 and observed OTU metrics. The beta diversity was calculated using weighted and unweighted UniFrac [22] distance measures and presented as principal coordinates analysis (PCoA) plots. Phylogenetic Investigation of Communities by Reconstruction of Unobserved States (PICRUSt) was used to infer functional capacity associated with taxonomic composition using Kyoto Encyclopedia of Genes and Genomes (KEGG) metabolic pathways after eliminating de-novo OTU [23]. Statistical analysis was conducted via Statistical Analyses of Metagenomic Profiles software 2.1.3 [24] using ANOVA and Tukey–Kramer multiple comparison tests. *P* values were adjusted for multiple inferences using the Benjamini–Hochberg method to control for false discovery rate of 0.05. Statistical significance was set at *p* < 0.05.

## Results

### Alpha and beta diversity measures

**Fig. 1:**
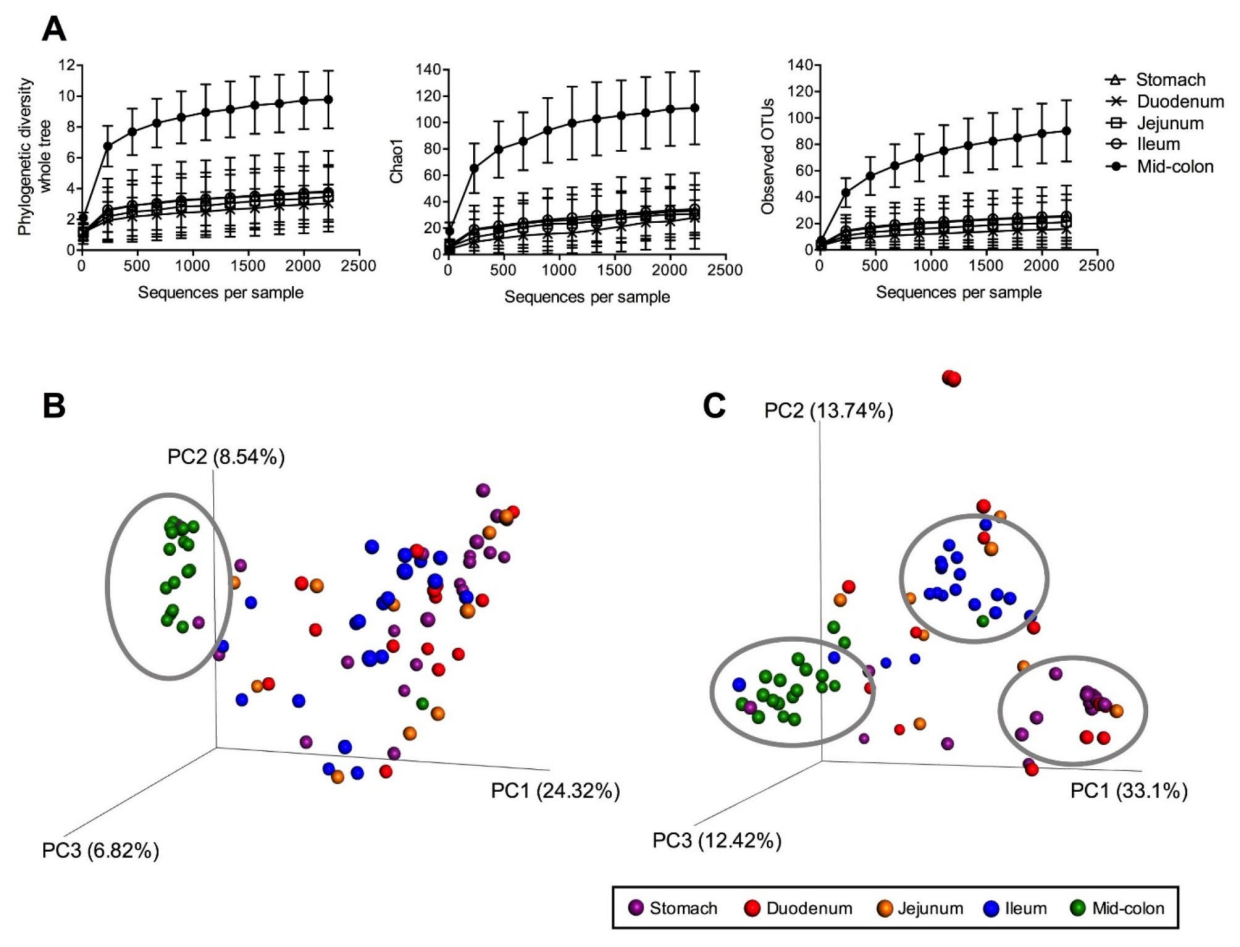
Alpha diversity measures (**A**), including phylogenetic diversity whole tree, Chao1, and observed operational taxonomic units (OTU) suggested that species richness and diversity were greater in the mid-colon segment than other segments (*p* = 0.010). Principal coordinates analysis (PCoA) plots of unweighted UniFrac distances (**B**) of mucosal microbial communities showed that mid-colon samples clustered together (circled area) and away from other samples. Weighted UniFrac distance (**C**) revealed distinct clusters of stomach, ileal, and mid-colon samples (circled areas)

The assessment of alpha diversity measures, including PD whole tree, Chao1, and observed OTU, revealed a higher species richness in the microbiota of the mid-colon samples (*p* = 0.010) than samples from other segments (Fig. 1A**)**. However, neither age (*p* = 0.322), sex (*p* = 0.645), nor diet (*p* = 0.856) demonstrated an impact on alpha diversity measures (Table 2).

**Table 2:** Alpha diversity measures including phylogenetic diversity (PD) whole tree, Chao 1, and observed operational taxonomic units (OTU) of mucosal microbiota in old and young dogs, dogs that consumed animal product-based (APB) and plant product-based (PPB) diets, and female and male dogs

Factors	PD whole tree	Chao1	Observed OTU
Age			
Old	5.35 ± 3.38	54.14 ± 41.19	41.97 ± 34.25
Young	4.60 ± 3.13	45.34 ± 38.77	33.83 ± 33.70
*p*-value	0.322	0.326	0.259
Diet			
APB	5.01 ± 3.34	49.86 ± 40.15	37.99 ± 34.30
PPB	4.90 ± 3.20	49.17 ± 40.22	37.39 ± 34.09
*p*-value	0.856	0.930	0.935
Sex			
Female	4.79 ± 3.16	47.42 ± 38.15	35.98 ± 32.08
Male	5.10 ± 3.36	51.38 ± 41.79	39.22 ± 35.88
*p*-value	0.645	0.670	0.663

Similar to our observations in alpha diversity, the PCoA plots of unweighted UniFrac distances clearly illustrated that the mid-colon samples formed a distinct cluster, which was separated from the samples from other segments (Fig. 1B). Furthermore, the PCoA plot using weighted UniFrac distances exhibited distinct clusters among the stomach, ileal, and mid-colon samples (Fig. 1C), signifying differential microbial compositions among these segments. Beta diversity was not different between age, sex, or diet groups as no distinct clusters were observed in the PCoA plots using both unweighted and weighted UniFrac distances (data not shown).

### Taxonomic composition

**Fig. 2:**
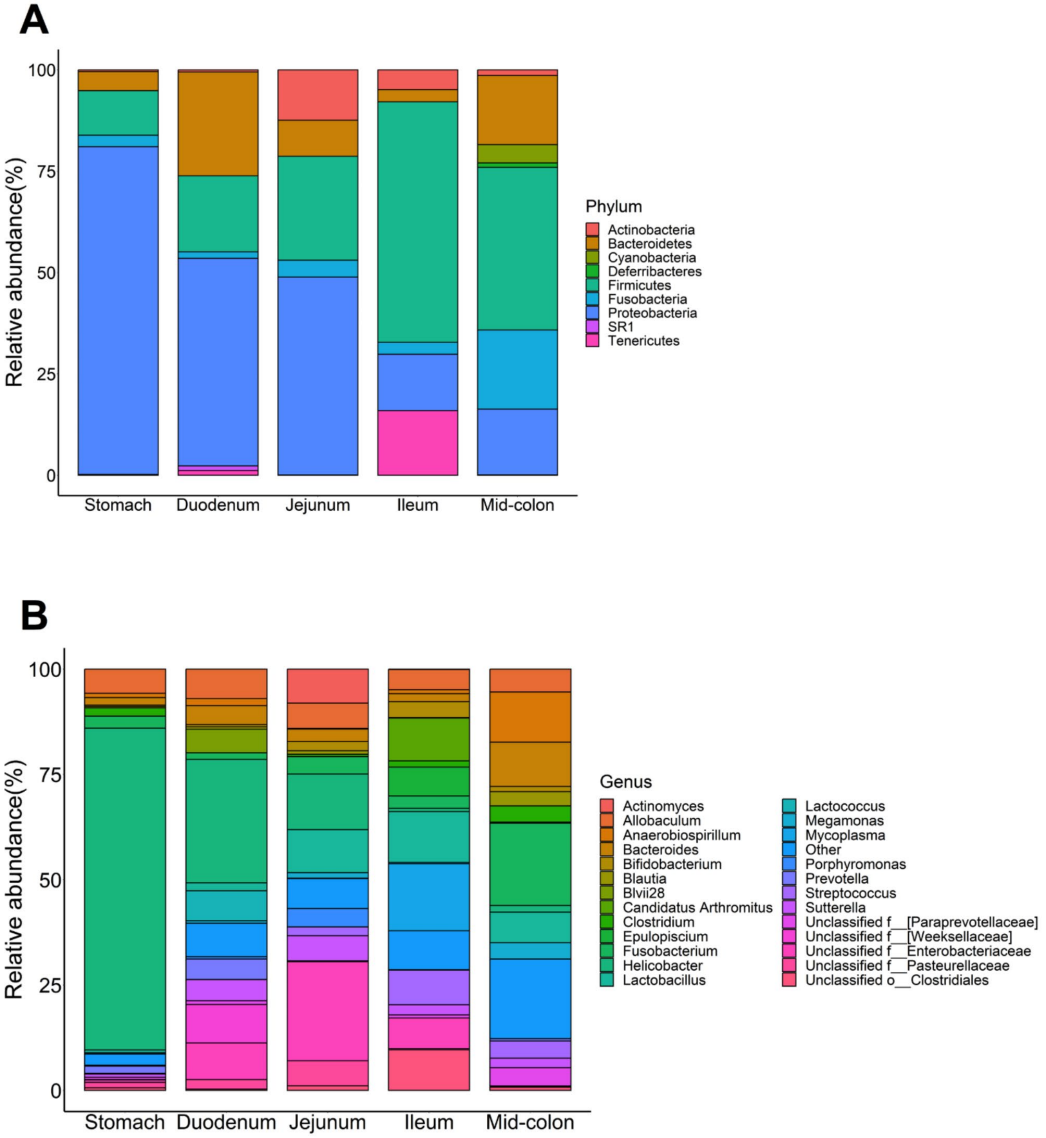
Relative abundances (% of sequences) of bacteria at the phylum (**A**) and genus (≥ 1%, **B**) levels of canine gastrointestinal tract segments

A comprehensive taxonomic summary of the GI tract segments at both the phylum and genus levels are presented in Table 3; Fig. 2. The predominant bacterial phyla observed were Proteobacteria (mean = 42.4%), Firmicutes (mean = 31.6%), Bacteroidetes (mean = 10.9%), and Fusobacteria (mean = 6.5%). These phyla exhibited significant variations between the different regions of the GI tract. The Cohen classified effect sizes were 0.03 to 0.6 depending on the taxonomies. Specifically, Proteobacteria had a decreasing trend from the stomach to the mid-colon (q < 0.001). The highest relative abundance of Firmicutes was measured in the ileum, while the lowest abundance was measured in the stomach (*q* < 0.001). In comparison, the duodenum had a higher relative abundance of Bacteroidetes than the stomach and ileum (*q* < 0.001). Moreover, Fusobacteria had a higher relative abundance in the mid-colon than the other regions (*q* < 0.001).

**Table 3:** Relative abundances (% of sequences) of bacterial phyla and genera of canine gastrointestinal tract segments

Taxon	Stomach	Duodenum	Jejunum	Ileum	Mid-colon	*p*-value	q-value
**Actinobacteria**	0.43 ± 0.71	0.53 ± 1.27	12.44 ± 29.10	4.9 ± 8.68	1.39 ± 2.89	0.050	0.064
* Actinomyces*	0.00 ± 0.01	0.00 ± 0.00	8.05 ± 24.16	0.07 ± 0.28	0.01 ± 0.02	0.106	0.211
* Bifidobacterium*	0.31 ± 0.66	0.53 ± 1.27	2.18 ± 3.48	3.77 ± 8.47	1.26 ± 2.89	0.140	0.252
* Brachybacterium*	0.05 ± 0.24	0.00 ± 0.00	1.82 ± 5.46	0.00 ± 0.00	0.00 ± 0.00	0.110	0.209
* Collinsella*	0.00 ± 0.00	0.00 ± 0.00	0.00 ± 0.00	0.04 ± 0.17	0.11 ± 0.36	0.350	0.450
* Corynebacterium*	0.06 ± 0.28	0.00 ± 0.01	0.00 ± 0.00	0.77 ± 2.19	0.01 ± 0.03	0.142	0.249
* Leucobacter*	0.00 ± 0.00	0.00 ± 0.00	0.38 ± 1.14	0.25 ± 1.09	0.00 ± 0.00	0.419	0.520
**Bacteroidetes**	4.70 ± 10.20^b^	25.58 ± 31.68^a^	8.88 ± 17.44^ab^	2.99 ± 9.38^b^	17.06 ± 6.66^ab^	< 0.001	< 0.001
* [Prevotella]*	0.25 ± 0.62	0.00 ± 0.01	1.30 ± 3.89	0.34 ± 1.42	1.51 ± 1.08	0.036	0.087
* Bacteroides*	1.78 ± 3.38^b^	4.49 ± 12.58^ab^	2.95 ± 8.71^ab^	1.89 ± 5.31^ab^	10.51 ± 4.08^a^	0.001	0.005
* Blvii28*	0.00 ± 0.00	5.61 ± 20.24	0.00 ± 0.00	0.00 ± 0.00	0.00 ± 0.00	0.276	0.405
* Parabacteroides*	0.06 ± 0.29	0.00 ± 0.00	0.00 ± 0.00	0.00 ± 0.00	0.02 ± 0.04	0.672	0.681
* Porphyromonas*	0.22 ± 0.59	0.53 ± 1.92	4.39 ± 12.6	0.00 ± 0.00	0.00 ± 0.00	0.106	0.206
* Prevotella*	1.71 ± 6.24	4.88 ± 15.29	0.02 ± 0.07	0.07 ± 0.28	0.54 ± 0.67	0.333	0.452
Unclassified f__[Paraprevotellaceae]	0.52 ± 1.94^b^	0.88 ± 2.97^b^	0.13 ± 0.38^b^	0.68 ± 2.89^b^	4.36 ± 3.37^a^	< 0.001	< 0.001
Unclassified f__[Weeksellaceae]	0.11 ± 0.34	9.13 ± 23.62	0.09 ± 0.27	0.00 ± 0.01	0.11 ± 0.47	0.050	0.112
Unclassified f__S24-7	0.06 ± 0.22	0.04 ± 0.16	0.00 ± 0.00	0.00 ± 0.00	0.02 ± 0.04	0.557	0.627
**Cyanobacteria**	0.00 ± 0.00	0.00 ± 0.00	0.00 ± 0.00	0.00 ± 0.00	4.52 ± 19.16	0.482	0.482
Unclassified o__Streptophyta	0.00 ± 0.00	0.00 ± 0.00	0.00 ± 0.00	0.00 ± 0.00	4.52 ± 19.16	0.482	0.578
**Deferribacteres**	0.03 ± 0.14^b^	0.00 ± 0.00^b^	0.00 ± 0.00^b^	0.00 ± 0.00^b^	1.10 ± 1.86^a^	< 0.001	< 0.001
* Mucispirillum*	0.03 ± 0.14^b^	0.00 ± 0.00^b^	0.00 ± 0.00^b^	0.00 ± 0.00^b^	1.10 ± 1.86^a^	0.001	0.003
**Firmicutes**	10.97 ± 20.00^c^	18.77 ± 28.60^bc^	25.65 ± 35.46^bc^	59.32 ± 30.47^a^	40.09 ± 14.77^ab^	< 0.001	< 0.001
* [Eubacterium]*	0.04 ± 0.16^b^	0.00 ± 0.00^b^	0.34 ± 0.97^ab^	0.03 ± 0.09^b^	1.17 ± 1.45^a^	< 0.001	< 0.001
* [Ruminococcus]*	0.10 ± 0.49^b^	0.00 ± 0.00^b^	0.42 ± 1.09^b^	0.08 ± 0.13^b^	1.56 ± 1.10^a^	< 0.001	< 0.001
* Allobaculum*	5.74 ± 17.10	6.96 ± 14.15	6.00 ± 14.97	4.83 ± 10.41	5.45 ± 7.68	0.995	0.995
* Anaerococcus*	0.11 ± 0.52	0.00 ± 0.00	0.00 ± 0.00	0.00 ± 0.00	0.00 ± 0.00	0.611	0.647
* Blautia*	0.32 ± 0.91^b^	0.56 ± 1.51^b^	0.84 ± 1.93^b^	0.13 ± 0.37^b^	3.35 ± 1.58^a^	< 0.001	< 0.001
* Candidatus Arthromitus*	0.00 ± 0.00	0.00 ± 0.00	0.01 ± 0.01	10.16 ± 23.86	0.02 ± 0.05	0.031	0.079
* Catenibacterium*	0.05 ± 0.16^b^	0.00 ± 0.00^b^	0.54 ± 1.58^b^	0.06 ± 0.28^ab^	0.87 ± 1.29^a^	0.009	0.031
* Clostridium*	1.99 ± 7.30	0.01 ± 0.01	0.55 ± 1.60	1.45 ± 2.69	3.88 ± 2.22	0.104	0.214
* Clostridium*_f__Lachnospiraceae	0.00 ± 0.00	0.00 ± 0.00	0.00 ± 0.00	0.09 ± 0.40	0.01 ± 0.03	0.531	0.607
* Coprobacillus*	0.01 ± 0.04^b^	0.00 ± 0.00^b^	0.00 ± 0.00^b^	0.00 ± 0.00^b^	0.05 ± 0.08^a^	0.004	0.013
* Dorea*	0.06 ± 0.20^b^	0.00 ± 0.00^b^	0.00 ± 0.00^b^	0.17 ± 0.73^ab^	0.57 ± 0.53^a^	0.002	0.007
* Enterococcus*	0.00 ± 0.00	0.00 ± 0.00	0.13 ± 0.38	0.24 ± 1.05	0.02 ± 0.03	0.559	0.619
* Epulopiscium*	0.04 ± 0.15	0.00 ± 0.00	0.00 ± 0.01	6.88 ± 21.88	0.23 ± 0.50	0.210	0.359
* Faecalibacterium*	0.26 ± 0.71^b^	0.01 ± 0.01^b^	0.65 ± 1.93^b^	0.02 ± 0.06	2.66 ± 2.41^a^	< 0.001	< 0.001
* Gemella*	0.01 ± 0.05	0.00 ± 0.00	0.00 ± 0.00	0.37 ± 0.86	0.01 ± 0.03	0.037	0.086
* Lactobacillus*	0.66 ± 2.08	1.87 ± 3.79	10.24 ± 15.16	12.08 ± 21.12	7.20 ± 10.62	0.035	0.086
* Lactococcus*	0.00 ± 0.00	7.12 ± 25.68	0.00 ± 0.00	0.00 ± 0.00	0.00 ± 0.00	0.276	0.413
* Megamonas*	0.26 ± 0.66^b^	0.58 ± 2.07^b^	1.29 ± 3.57^ab^	0.28 ± 0.64^b^	3.93 ± 4.36^a^	< 0.001	0.001
* Peptococcus*	0.00 ± 0.01	0.00 ± 0.00	0.02 ± 0.03	0.15 ± 0.47	0.14 ± 0.27	0.228	0.356
* Phascolarctobacterium*	0.05 ± 0.16^b^	0.00 ± 0.01^b^	0.11 ± 0.21^b^	0.11 ± 0.38^b^	0.70 ± 0.46^a^	< 0.001	< 0.001
* Roseburia*	0.00 ± 0.01^b^	0.00 ± 0.00^b^	0.00 ± 0.00^b^	0.00 ± 0.00^b^	0.05 ± 0.09^a^	0.001	0.004
* Staphylococcus*	0.02 ± 0.05	0.00 ± 0.00	0.00 ± 0.00	0.00 ± 0.00	0.00 ± 0.00	0.227	0.363
* Streptococcus*	0.17 ± 0.44^b^	0.06 ± 0.18^b^	2.06 ± 4.76^ab^	8.25 ± 14.46^a^	4.07 ± 7.02^ab^	0.014	0.041
* Turicibacter*	0.23 ± 0.67	0.05 ± 0.17	1.18 ± 2.12	0.49 ± 1.26	0.21 ± 0.41	0.094	0.198
* Veillonella*	0.00 ± 0.00	0.08 ± 0.27	0.13 ± 0.39	0.24 ± 0.97	0.05 ± 0.11	0.627	0.645
Unclassified f__Aerococcaceae	0.00 ± 0.01^b^	0.00 ± 0.00^b^	0.00 ± 0.00^b^	1.58 ± 3.47^a^	0.03 ± 0.08^b^	0.017	0.049
Unclassified f__Clostridiaceae	0.02 ± 0.07^b^	0.00 ± 0.00^b^	0.01 ± 0.01^b^	0.07 ± 0.12^b^	0.25 ± 0.19^a^	< 0.001	< 0.001
Unclassified f__Erysipelotrichaceae	0.01 ± 0.03^b^	0.00 ± 0.00^b^	0.00 ± 0.01^b^	0.00 ± 0.00^b^	0.19 ± 0.25^a^	< 0.001	< 0.001
Unclassified f__Lachnospiraceae	0.11 ± 0.36^b^	0.06 ± 0.18^b^	0.04 ± 0.13^b^	0.05 ± 0.16^b^	1.29 ± 1.28^a^	< 0.001	< 0.001
Unclassified f__Peptostreptococcaceae	0.03 ± 0.11	0.00 ± 0.01	0.01 ± 0.01	1.75 ± 3.68	0.39 ± 0.69	0.020	0.056
Unclassified f__Ruminococcaceae	0.04 ± 0.15^b^	1.12 ± 2.76^a^	0.00 ± 0.00^b^	0.12 ± 0.35^b^	1.00 ± 0.66^a^	0.012	0.038
Unclassified o__Clostridiales	0.62 ± 1.06^b^	0.29 ± 0.68^b^	1.09 ± 2.88^b^	9.64 ± 13.00^a^	0.77 ± 0.76^b^	< 0.001	< 0.001
**Fusobacteria**	2.86 ± 8.24^b^	1.60 ± 4.01^b^	4.15 ± 8.71^b^	2.94 ± 7.67^b^	19.54 ± 12.32^a^	< 0.001	< 0.001
* Fusobacterium*	2.82 ± 8.24^b^	1.60 ± 4.01^b^	4.15 ± 8.71^b^	2.94 ± 7.67^b^	19.54 ± 12.32^a^	< 0.001	< 0.001
Unclassified f__Leptotrichiaceae	0.04 ± 0.17	0.00 ± 0.00	0.00 ± 0.00	0.00 ± 0.00	0.00 ± 0.00	0.610	0.656
**Proteobacteria**	80.79 ± 29.29^a^	51.17 ± 39.78^b^	48.77 ± 40.51^b^	13.9 ± 21.58^c^	16.19 ± 11.56^c^	< 0.001	< 0.001
* Acinetobacter*	0.06 ± 0.27	0.00 ± 0.00	0.00 ± 0.00	0.27 ± 1.17	0 ± 0.01	0.605	0.660
* Anaerobiospirillum*	1.00 ± 2.87^b^	1.70 ± 4.99^b^	0.17 ± 0.48	0.94 ± 3.16^b^	11.87 ± 11.36^a^	< 0.001	< 0.001
* Campylobacter*	0.00 ± 0.01	1.47 ± 5.29	0.01 ± 0.03	0.00 ± 0.00	0.10 ± 0.26	0.294	0.423
* Helicobacter*	76.38 ± 31.6^a^	29.27 ± 40.44^b^	13.17 ± 27.28^bc^	0.76 ± 2.33^c^	1.59 ± 2.22^c^	< 0.001	< 0.001
* Moraxella*	0.03 ± 0.09	0.00 ± 0.00	0.00 ± 0.00	0.05 ± 0.17	0.00 ± 0.00	0.428	0.522
* Pasteurella*	0.09 ± 0.33	0.40 ± 1.43	0.00 ± 0.00	0.00 ± 0.00	0.00 ± 0.00	0.356	0.450
* Psychrobacter*	0.00 ± 0.00	0.00 ± 0.00	0.00 ± 0.00	1.60 ± 6.98	0.00 ± 0.00	0.517	0.611
* Sutterella*	0.77 ± 2.03	4.98 ± 12.36	5.94 ± 14.51	2.42 ± 4.35	2.28 ± 1.99	0.348	0.455
Unclassified f__Bradyrhizobiaceae	0.00 ± 0.01	0.00 ± 0.00	0.00 ± 0.00	0.26 ± 1.11	0.00 ± 0.00	0.519	0.603
Unclassified f__Comamonadaceae	0.00 ± 0.00	1.32 ± 4.75	0.00 ± 0.00	0.00 ± 0.00	0.00 ± 0.00	0.273	0.418
Unclassified f__Enterobacteriaceae	0.58 ± 1.21	8.67 ± 25.33	23.55 ± 38.72	7.28 ± 18.06	0.16 ± 0.30	0.023	0.061
Unclassified f__Helicobacteraceae	0.01 ± 0.05	0.00 ± 0.00	0.00 ± 0.00	0.00 ± 0.00	0.05 ± 0.11	0.073	0.160
Unclassified f__Moraxellaceae	0.14 ± 0.43	0.00 ± 0.00	0.00 ± 0.00	0.00 ± 0.02	0.00 ± 0.00	0.218	0.365
Unclassified f__Neisseriaceae	0.31 ± 0.65	1.06 ± 3.82	0.00 ± 0.00	0.00 ± 0.00	0.01 ± 0.05	0.337	0.450
Unclassified f__Pasteurellaceae	1.30 ± 2.79	2.32 ± 7.24	5.93 ± 17.17	0.31 ± 1.01	0.01 ± 0.02	0.226	0.370
Unclassified f__Succinivibrionaceae	0.01 ± 0.06	0.00 ± 0.00	0.00 ± 0.00	0.00 ± 0.00	0.12 ± 0.33	0.127	0.235
Unclassified f__Xanthomonadaceae	0.09 ± 0.42	0.00 ± 0.00	0.00 ± 0.00	0.00 ± 0.00	0.00 ± 0.00	0.614	0.641
**Tenericutes**	0.15 ± 0.63^c^	1.21 ± 4.34^b^	0.11 ± 0.31^b^	15.96 ± 26.88^a^	0.13 ± 0.11^b^	< 0.001	< 0.001
* Anaeroplasma*	0.01 ± 0.05^b^	0.00 ± 0.01^b^	0.00 ± 0.00^b^	0.00 ± 0.01^b^	0.12 ± 0.10^a^	< 0.001	< 0.001
* Mycoplasma*	0.07 ± 0.32^b^	0.00 ± 0.00^b^	0.11 ± 0.31^b^	15.95 ± 26.88^a^	0.01 ± 0.03^b^	0.001	0.003
* Ureaplasma*	0.07 ± 0.31	1.20 ± 4.34	0.00 ± 0.00	0.00 ± 0.00	0.00 ± 0.00	0.295	0.417
**SR1**	0.08 ± 0.36	1.13 ± 4.07	0.00 ± 0.00	0.00 ± 0.00	0.00 ± 0.00	0.301	0.338
Unclassified p__SR1	0.08 ± 0.36	1.13 ± 4.07	0.00 ± 0.00	0.00 ± 0.00	0.00 ± 0.00	0.301	0.416

At the genus level, the relative abundances of more than 20 genera were shown to be different across the different GI segments, including *Bacteroides*, *Blautia*, *Faecalibacterium*, *Megamonas*, and *Streptococcus*. The relative abundances of taxa at various levels were not influenced by diet, sex, or age. The Cohen classified effect sizes for these factors were below 0.2 suggesting that the sample size might not provide sufficient statistical power to detect the biological differences.

### Functional capacity

At the L1 hierarchy of the PICRUSt results, the predicted functional capacity of mucosal microbiota from the five GI tract segments were primarily categorized into three main pathway categories: metabolism (mean = 49.2%), genetic information processing (mean = 23.3%), and environmental information processing (mean = 12.3%). Notably, all L1 pathway categories exhibited significant differences among the GI tract segments (*q* < 0.05; Table 4). Specifically, the functions related to environmental information processing were shown to be lower in the stomach and duodenum compared with the other segments (*q* < 0.001). The stomach displayed greater potential in cellular processes compared with that of the other segments (*q* < 0.001), while the capacities related to human diseases decreased from the stomach to the mid-colon (*q* < 0.001).

**Table 4:** Predicted bacterial functional pathway (KEGG pathways at L1 and L2 hierarchies) abundances of canine gastrointestinal tract segments

Pathway Category	Stomach	Duodenum	Jejunum	Ileum	Mid-colon	*p*-value	q-value
**Cellular processes**	6.818 ± 1.949^a^	3.636 ± 2.552^b^	2.841 ± 1.676^b^	2.916 ± 1.525^b^	2.168 ± 0.403^b^	< 0.001	< 0.001
Cell growth and death	0.699 ± 0.076^a^	0.535 ± 0.113^b^	0.482 ± 0.103^b^	0.505 ± 0.064^b^	0.541 ± 0.027^b^	< 0.001	< 0.001
Cell motility	5.923 ± 1.881^a^	2.795 ± 2.587^b^	2.107 ± 1.761^b^	2.253 ± 1.544^b^	1.420 ± 0.401^b^	< 0.001	< 0.001
Transport and catabolism	0.195 ± 0.026^bc^	0.306 ± 0.142^a^	0.252 ± 0.092^ab^	0.158 ± 0.076^c^	0.207 ± 0.019^bc^	< 0.001	< 0.001
**Cellular processes and signaling**	3.712 ± 0.172^ab^	4.067 ± 0.664^a^	4.180 ± 0.961^a^	3.393 ± 0.779^b^	3.875 ± 0.236^ab^	0.004	0.005
Cell division	0.017 ± 0.025^c^	0.073 ± 0.042^ab^	0.079 ± 0.033^ab^	0.050 ± 0.024^b^	0.080 ± 0.011^a^	< 0.001	< 0.001
Cell motility and secretion	0.484 ± 0.124^a^	0.294 ± 0.134^b^	0.247 ± 0.098^bc^	0.168 ± 0.041^c^	0.195 ± 0.027^c^	< 0.001	< 0.001
Electron transfer carriers	0.053 ± 0.015	0.05 ± 0.051	0.069 ± 0.066^a^	0.029 ± 0.035	0.027 ± 0.012^b^	0.013	0.016
Germination	0.003 ± 0.006^c^	0.002 ± 0.005^c^	0.005 ± 0.008^bc^	0.017 ± 0.024^ab^	0.022 ± 0.008^a^	< 0.001	< 0.001
Inorganic ion transport and metabolism	0.232 ± 0.037^a^	0.265 ± 0.114^a^	0.270 ± 0.134^a^	0.204 ± 0.079^ab^	0.150 ± 0.014^b^	< 0.001	< 0.001
Membrane and intracellular structural molecules	0.762 ± 0.109^a^	0.758 ± 0.175^a^	0.675 ± 0.290^ab^	0.393 ± 0.183^c^	0.511 ± 0.060^bc^	< 0.001	< 0.001
Other ion-coupled transporters	1.169 ± 0.065^bc^	1.358 ± 0.201^ab^	1.477 ± 0.237^a^	1.082 ± 0.338^c^	1.296 ± 0.113^ab^	< 0.001	< 0.001
Other transporters	0.157 ± 0.046^b^	0.26 ± 0.094^a^	0.284 ± 0.078^a^	0.223 ± 0.077^a^	0.265 ± 0.018^a^	< 0.001	< 0.001
Pores ion channels	0.318 ± 0.044^bc^	0.527 ± 0.244^a^	0.460 ± 0.236^ab^	0.249 ± 0.135^c^	0.324 ± 0.082^bc^	< 0.001	< 0.001
Signal transduction mechanisms	0.348 ± 0.056^c^	0.405 ± 0.144^bc^	0.489 ± 0.141^ab^	0.506 ± 0.072^a^	0.501 ± 0.044^a^	< 0.001	< 0.001
Sporulation	0.169 ± 0.121^b^	0.076 ± 0.078^b^	0.126 ± 0.182^b^	0.473 ± 0.403^a^	0.504 ± 0.158^a^	< 0.001	< 0.001
**Environmental information processing**	11.055 ± 1.351^b^	11.862 ± 2.972^b^	14.320 ± 2.589^a^	15.302 ± 1.630^a^	14.280 ± 0.422^a^	< 0.001	< 0.001
Membrane transport	8.384 ± 1.725^b^	9.799 ± 2.721^b^	12.228 ± 2.140^a^	13.526 ± 1.365^a^	12.575 ± 0.529^a^	< 0.001	< 0.001
Signal transduction	2.475 ± 0.448^a^	1.856 ± 0.693^b^	1.911 ± 0.733^b^	1.570 ± 0.487^b^	1.504 ± 0.213^b^	< 0.001	< 0.001
Signaling molecules and interaction	0.197 ± 0.029	0.206 ± 0.078	0.182 ± 0.083	0.206 ± 0.080	0.201 ± 0.031	0.877	0.921
**Genetic information processing**	22.422 ± 0.657^b^	22.997 ± 2.153^ab^	22.821 ± 2.352^ab^	25.096 ± 4.160^a^	23.154 ± 1.023^ab^	0.011	0.011
Folding, sorting and degradation	3.254 ± 0.303^a^	2.858 ± 0.407^b^	2.696 ± 0.335^b^	2.579 ± 0.418^b^	2.529 ± 0.112^b^	< 0.001	< 0.001
Protein folding and associated processing	1.130 ± 0.182^a^	0.842 ± 0.212^b^	0.776 ± 0.120^bc^	0.591 ± 0.092^d^	0.646 ± 0.091^cd^	< 0.001	< 0.001
Replication and repair	7.912 ± 0.773^b^	8.948 ± 1.348^ab^	9.103 ± 1.240^ab^	10.115 ± 1.761^a^	9.407 ± 0.593^a^	< 0.001	< 0.001
Replication, recombination and repair proteins	0.673 ± 0.075	0.887 ± 0.565	0.891 ± 0.165	0.837 ± 0.151	0.879 ± 0.062	0.045	0.053
Restriction enzyme	0.248 ± 0.027	0.209 ± 0.081	0.183 ± 0.102	0.227 ± 0.112	0.197 ± 0.032	0.123	0.136
Transcription	1.463 ± 0.415^c^	2.031 ± 0.470^c^	2.320 ± 0.362^ab^	2.654 ± 0.284^a^	2.572 ± 0.211^a^	< 0.001	< 0.001
Transcription related proteins	0.001 ± 0.002^b^	0.015 ± 0.025^a^	0.015 ± 0.012^ab^	0.013 ± 0.012^a^	0.008 ± 0.005^ab^	0.008	0.010
Translation	6.850 ± 0.257^ab^	6.280 ± 0.920^ab^	5.931 ± 1.092^ab^	7.107 ± 2.064^a^	5.983 ± 0.399^b^	0.012	0.015
Translation proteins	0.891 ± 0.023^b^	0.926 ± 0.088^ab^	0.907 ± 0.102^ab^	0.973 ± 0.103^a^	0.934 ± 0.026^ab^	0.012	0.015
**Human diseases**	1.700 ± 0.365^a^	1.151 ± 0.385^b^	0.979 ± 0.252^bc^	0.905 ± 0.185^bc^	0.776 ± 0.096^c^	< 0.001	< 0.001
Cancers	0.227 ± 0.037^a^	0.150 ± 0.061^b^	0.142 ± 0.037^bc^	0.098 ± 0.032^d^	0.108 ± 0.016^cd^	< 0.001	< 0.001
Cardiovascular diseases	0.000 ± 0.000	0.000 ± 0.000	0.000 ± 0.000	0.001 ± 0.003	0.000 ± 0.000	0.188	0.201
Immune system diseases	0.061 ± 0.004	0.061 ± 0.013	0.066 ± 0.019	0.069 ± 0.019	0.058 ± 0.008	0.108	0.121
Infectious diseases	0.675 ± 0.115^a^	0.473 ± 0.149^b^	0.436 ± 0.151^b^	0.463 ± 0.084^b^	0.404 ± 0.074^b^	< 0.001	< 0.001
Metabolic diseases	0.120 ± 0.007^a^	0.099 ± 0.027^ab^	0.093 ± 0.018^b^	0.122 ± 0.040^a^	0.100 ± 0.016^ab^	0.002	0.003
Neurodegenerative diseases	0.618 ± 0.208^a^	0.368 ± 0.275^b^	0.242 ± 0.221^bc^	0.151 ± 0.072^c^	0.106 ± 0.026^c^	< 0.001	< 0.001
**Metabolism**	49.369 ± 0.595^a^	50.561 ± 2.655^a^	49.019 ± 1.797^ab^	46.987 ± 2.630^b^	50.212 ± 1.023^a^	< 0.001	< 0.001
Amino acid metabolism	8.681 ± 0.277^bc^	9.547 ± 1.011^a^	9.064 ± 0.685^abc^	8.431 ± 0.966^c^	9.269 ± 0.325^ab^	< 0.001	< 0.001
Biosynthesis and biodegradation of secondary metabolites	0.060 ± 0.005^ab^	0.076 ± 0.029^a^	0.074 ± 0.047^ab^	0.034 ± 0.028^c^	0.048 ± 0.01^bc^	< 0.001	< 0.001
Biosynthesis of other secondary metabolites	0.473 ± 0.160^c^	0.787 ± 0.282^ab^	0.674 ± 0.154^ab^	0.628 ± 0.196^bc^	0.872 ± 0.073^a^	< 0.001	< 0.001
Carbohydrate metabolism	8.798 ± 0.746^b^	9.510 ± 1.097^b^	9.749 ± 1.098^ab^	10.062 ± 0.753^a^	10.655 ± 0.558^a^	< 0.001	< 0.001
Energy metabolism	7.659 ± 0.679^a^	6.404 ± 0.989^b^	5.839 ± 0.624^bc^	5.536 ± 0.468^c^	6.069 ± 1.015^bc^	< 0.001	< 0.001
Energy metabolism	0.728 ± 0.068^b^	0.839 ± 0.142^ab^	0.857 ± 0.201^ab^	0.707 ± 0.177^b^	0.854 ± 0.115^a^	0.002	0.003
Enzyme families	1.867 ± 0.220^b^	2.036 ± 0.270^ab^	2.138 ± 0.188^a^	2.119 ± 0.196^a^	2.177 ± 0.190^ab^	< 0.001	< 0.001
Glycan biosynthesis and metabolism	3.156 ± 0.395^a^	2.990 ± 0.521^a^	2.622 ± 0.656^ab^	1.696 ± 0.698^c^	2.305 ± 0.170^bc^	< 0.001	< 0.001
Lipid metabolism	2.696 ± 0.173	2.748 ± 0.385	2.659 ± 0.205	2.717 ± 0.292	2.694 ± 0.089	0.928	0.958
Metabolism of cofactors and vitamins	4.738 ± 0.317^a^	4.467 ± 0.463^a^	4.442 ± 0.565^a^	3.640 ± 0.473^b^	4.509 ± 0.396^a^	< 0.001	< 0.001
Metabolism of other amino acids	1.743 ± 0.108^a^	1.642 ± 0.156^ab^	1.526 ± 0.098^bc^	1.512 ± 0.098^c^	1.446 ± 0.047^c^	< 0.001	< 0.001
Metabolism of terpenoids and polyketides	1.744 ± 0.033^ab^	1.812 ± 0.202^a^	1.684 ± 0.162^ab^	1.643 ± 0.152^b^	1.685 ± 0.104^ab^	0.008	0.010
Nucleotide metabolism	0.016 ± 0.030^c^	0.038 ± 0.030^bc^	0.073 ± 0.048^ab^	0.076 ± 0.035^a^	0.051 ± 0.019^ab^	< 0.001	< 0.001
Nucleotide metabolism	4.477 ± 0.087	4.311 ± 0.532	4.290 ± 0.614	4.616 ± 0.688	4.270 ± 0.287	0.154	0.167
Others	0.642 ± 0.140^b^	0.953 ± 0.500^a^	0.932 ± 0.183^a^	1.087 ± 0.145^a^	0.965 ± 0.111^a^	< 0.001	< 0.001
Xenobiotics biodegradation and metabolism	1.368 ± 0.094^b^	1.843 ± 0.618^a^	1.784 ± 0.383^a^	1.843 ± 0.402^a^	1.689 ± 0.135^a^	< 0.001	< 0.001
**Organismal systems**	0.833 ± 0.066^a^	0.710 ± 0.134^b^	0.664 ± 0.182^bc^	0.565 ± 0.111^c^	0.642 ± 0.064^bc^	< 0.001	< 0.001
Circulatory system	0.150 ± 0.058^a^	0.058 ± 0.075^b^	0.024 ± 0.049^bc^	0.005 ± 0.010^c^	0.004 ± 0.006^c^	< 0.001	< 0.001
Digestive system	0.007 ± 0.016^b^	0.043 ± 0.047^a^	0.031 ± 0.027^ab^	0.024 ± 0.024^ab^	0.027 ± 0.025^ab^	0.007	0.009
Endocrine system	0.203 ± 0.027	0.262 ± 0.080	0.287 ± 0.161	0.207 ± 0.070	0.270 ± 0.051	0.006	0.008
Environmental adaptation	0.280 ± 0.048^a^	0.177 ± 0.076^b^	0.149 ± 0.050^b^	0.183 ± 0.065^b^	0.151 ± 0.020^b^	< 0.001	< 0.001
Excretory system	0.002 ± 0.004^c^	0.007 ± 0.011^bc^	0.020 ± 0.018^a^	0.013 ± 0.008^ab^	0.010 ± 0.006^abc^	< 0.001	< 0.001
Immune system	0.118 ± 0.009^a^	0.089 ± 0.025^b^	0.070 ± 0.027^bc^	0.053 ± 0.020^c^	0.084 ± 0.009^b^	< 0.001	< 0.001
Nervous system	0.073 ± 0.021	0.072 ± 0.026	0.083 ± 0.044	0.079 ± 0.027	0.095 ± 0.011	0.060	0.069
Function unknown	1.007 ± 0.081^c^	1.459 ± 0.410^ab^	1.682 ± 0.430^a^	1.335 ± 0.256^b^	1.279 ± 0.054^b^	< 0.001	< 0.001
General function prediction only	3.083 ± 0.375^a^	3.558 ± 0.471^b^	3.494 ± 0.347^b^	3.501 ± 0.191^b^	3.614 ± 0.132^b^	< 0.001	< 0.001

^a, b, c^ mean values within a row with unlike superscript letters are significantly different after (*q* < 0.05)

Within the environmental information processing pathway category, significance was mainly driven by the pathway category of membrane transport (*q* < 0.001). In the cellular processes pathway category, significance was driven by pathway categories such as cell growth and death, and cell motility (all *q* < 0.001). Regarding human diseases, significance was driven by pathway categories such as cancers, infectious diseases, and neurodegenerative diseases (all *q* < 0.001).

At the L3 hierarchy, a total of 200 pathway categories exhibited significant differences among the GI tract segments (Supplementary Table 1). We observed differences (*q* < 0.05) in bile acid-related pathway categories across the GI tract segments, accompanied by specific KEGG orthology (KO) alterations within the categories (Table 5). Specifically, enzymes involved in bile acid synthesis exhibited significant variations across the GI tract regions. Choloylglycine hydrolase (K01442) displayed significant variation (*q* = 0.005), while 7-alpha-hydroxysteroid dehydrogenase (K00076) and 3-dehydro-bile acid delta 4,6-reductase (K07007) showed highly significant differences (*q* < 0.001). Flagellar assembly pathways were also highly different among GI tract segments (Table 6). Stomach samples had the highest capacity among all segments, driven primarily by 31 altered KOs (q < 0.05) associated with this pathway. Predicted bacterial functional capacity of the mucosal microbiota of dogs was not influenced by diet, age, or sex.

**Table 5:** Significantly altered KEGG Orthology relative abundances associated with bile acid synthesis across canine GI tract segments

KEGG Orthology	Stomach	Duodenum	Jejunum	Ileum	Mid-colon	*p*-value	q-value
K01442: choloylglycine hydrolase [EC:3.5.1.24]	0.009 ± 0.019^b^	0.034 ± 0.027^a^	0.033 ± 0.026^ab^	0.029 ± 0.028^ab^	0.039 ± 0.015^a^	0.001	0.005
K00076: 7-alpha-hydroxysteroid dehydrogenase [EC:1.1.1.159]	0.088 ± 0.032^a^	0.035 ± 0.043^b^	0.018 ± 0.03^bc^	0.003 ± 0.006^c^	0.016 ± 0.007^bc^	< 0.001	< 0.001
K07007: 3-dehydro-bile acid delta 4,6-reductase [EC: 1.3.1.114]	0.017 ± 0.026^b^	0.037 ± 0.028^b^	0.034 ± 0.022^b^	0.065 ± 0.028^a^	0.071 ± 0.011^a^	< 0.001	< 0.001

^a, b, c^ mean values within a row with unlike superscript letters are significantly different (*q* < 0.05)

**Table 6:** Significantly altered KEGG Orthology relative abundances associated with flagellar assembly pathways across canine GI tract segments

KEGG Orthology	Stomach	Duodenum	Jejunum	Ileum	Mid-colon	*p*-value	q-value
K02386: flagella basal body P-ring formation protein FlgA	0.087 ± 0.034^a^	0.036 ± 0.044^b^	0.023 ± 0.030^bc^	0.005 ± 0.009^c^	0.004 ± 0.005^c^	< 0.001	< 0.001
K02387: flagellar basal-body rod protein FlgB	0.089 ± 0.033^a^	0.037 ± 0.043^b^	0.025 ± 0.030^b^	0.030 ± 0.027^b^	0.009 ± 0.007^b^	< 0.001	< 0.001
K02388: flagellar basal-body rod protein FlgC	0.089 ± 0.033^a^	0.037 ± 0.043^b^	0.025 ± 0.030^b^	0.030 ± 0.027^b^	0.009 ± 0.007^b^	< 0.001	< 0.001
K02389: flagellar basal-body rod modification protein FlgD	0.089 ± 0.033^a^	0.037 ± 0.043^b^	0.025 ± 0.030^bc^	0.022 ± 0.015^bc^	0.009 ± 0.007^c^	< 0.001	< 0.001
K02390: flagellar hook protein FlgE	0.175 ± 0.067^a^	0.068 ± 0.087^b^	0.038 ± 0.056^bc^	0.022 ± 0.032^bc^	0.010 ± 0.009^c^	< 0.001	< 0.001
K02392: flagellar basal-body rod protein FlgG	0.178 ± 0.064^a^	0.070 ± 0.085^b^	0.040 ± 0.056^bc^	0.046 ± 0.048^bc^	0.025 ± 0.012^c^	< 0.001	< 0.001
K02393: flagellar L-ring protein precursor FlgH	0.088 ± 0.033^a^	0.037 ± 0.043^b^	0.024 ± 0.029^bc^	0.005 ± 0.009^c^	0.006 ± 0.006^c^	< 0.001	< 0.001
K02394: flagellar P-ring protein precursor FlgI	0.088 ± 0.033^a^	0.037 ± 0.043^b^	0.024 ± 0.029^bc^	0.005 ± 0.009^c^	0.006 ± 0.006^c^	< 0.001	< 0.001
K02396: flagellar hook-associated protein 1 FlgK	0.088 ± 0.033^a^	0.036 ± 0.044^b^	0.024 ± 0.029^b^	0.025 ± 0.035^b^	0.010 ± 0.006^b^	< 0.001	< 0.001
K02397: flagellar hook-associated protein 3 FlgL	0.088 ± 0.034^a^	0.036 ± 0.044^b^	0.024 ± 0.030^bc^	0.017 ± 0.025^bc^	0.004 ± 0.004^c^	< 0.001	< 0.001
K02400: flagellar biosynthesis protein FlhA	0.089 ± 0.033^a^	0.037 ± 0.043^b^	0.025 ± 0.030^b^	0.030 ± 0.027^b^	0.009 ± 0.007^b^	< 0.001	< 0.001
K02401: flagellar biosynthetic protein FlhB	0.088 ± 0.033^a^	0.037 ± 0.043^b^	0.025 ± 0.029^bc^	0.009 ± 0.014^bc^	0.007 ± 0.006^c^	< 0.001	< 0.001
K02405: RNA polymerase sigma factor for flagellar operon FliA	0.089 ± 0.032^a^	0.037 ± 0.043^b^	0.026 ± 0.029^b^	0.029 ± 0.026^b^	0.011 ± 0.007^b^	< 0.001	< 0.001
K02406: flagellin	0.178 ± 0.063^a^	0.086 ± 0.104^b^	0.054 ± 0.081^b^	0.062 ± 0.086^b^	0.032 ± 0.016^b^	< 0.001	< 0.001
K02407: flagellar hook-associated protein 2	0.088 ± 0.033^a^	0.036 ± 0.044^b^	0.024 ± 0.029^b^	0.020 ± 0.028^b^	0.009 ± 0.006^b^	< 0.001	< 0.001
K02409: flagellar M-ring protein FliF	0.089 ± 0.033^a^	0.037 ± 0.043^b^	0.025 ± 0.030^b^	0.027 ± 0.023^b^	0.008 ± 0.007^b^	< 0.001	< 0.001
K02410: flagellar motor switch protein FliG	0.089 ± 0.033^a^	0.037 ± 0.043^b^	0.025 ± 0.030^b^	0.030 ± 0.027^b^	0.009 ± 0.007^b^	< 0.001	< 0.001
K02411: flagellar assembly protein FliH	0.089 ± 0.033^a^	0.037 ± 0.043^b^	0.025 ± 0.030^bc^	0.021 ± 0.015^bc^	0.008 ± 0.007^c^	< 0.001	< 0.001
K02412: flagellum-specific ATP synthase [EC:3.6.3.14]	0.089 ± 0.033^a^	0.037 ± 0.043^b^	0.025 ± 0.030^b^	0.030 ± 0.027^b^	0.009 ± 0.007^b^	< 0.001	< 0.001
K02413: flagellar FliJ protein	0.002 ± 0.005^b^	0.005 ± 0.011^b^	0.011 ± 0.016^ab^	0.019 ± 0.015^a^	0.004 ± 0.003^b^	< 0.001	< 0.001
K02414: flagellar hook-length control protein FliK	0.000 ± 0.001^b^	0.005 ± 0.011^ab^	0.010 ± 0.016^ab^	0.01 ± 0.015^a^	0.002 ± 0.003^ab^	0.018	0.049
K02415: flagellar FliL protein	0.088 ± 0.034^a^	0.036 ± 0.044^b^	0.024 ± 0.030^bc^	0.017 ± 0.025^bc^	0.005 ± 0.005^c^	< 0.001	< 0.001
K02416: flagellar motor switch protein FliM	0.089 ± 0.033^a^	0.037 ± 0.043^b^	0.025 ± 0.030^b^	0.030 ± 0.027^b^	0.009 ± 0.007^b^	< 0.001	< 0.001
K02417: flagellar motor switch protein FliN/FliY	0.177 ± 0.065^a^	0.069 ± 0.086^b^	0.040 ± 0.056^b^	0.036 ± 0.031^b^	0.018 ± 0.010^b^	< 0.001	< 0.001
K02418: flagellar protein FliO/FliZ	0.000 ± 0.001^b^	0.005 ± 0.011^ab^	0.010 ± 0.016^ab^	0.019 ± 0.025^a^	0.002 ± 0.001^b^	0.001	0.003
K02419: flagellar biosynthetic protein FliP	0.089 ± 0.033^a^	0.037 ± 0.043^b^	0.025 ± 0.030^b^	0.030 ± 0.027^b^	0.009 ± 0.007^b^	< 0.001	< 0.001
K02420: flagellar biosynthetic protein FliQ	0.089 ± 0.033^a^	0.037 ± 0.043^b^	0.025 ± 0.030^b^	0.030 ± 0.027^b^	0.009 ± 0.007^b^	< 0.001	< 0.001
K02421: flagellar biosynthetic protein FliR	0.088 ± 0.033^a^	0.037 ± 0.043^b^	0.025 ± 0.029^bc^	0.009 ± 0.014^bc^	0.007 ± 0.006^c^	< 0.001	< 0.001
K02422: flagellar protein FliS	0.090 ± 0.030^a^	0.037 ± 0.043^b^	0.026 ± 0.029^b^	0.039 ± 0.032^b^	0.02 ± 0.008^b^	< 0.001	< 0.001
K02556: chemotaxis protein MotA	0.090 ± 0.031^a^	0.035 ± 0.041^b^	0.025 ± 0.028^b^	0.038 ± 0.033^b^	0.016 ± 0.008^b^	< 0.001	< 0.001
K02557: chemotaxis protein MotB	0.093 ± 0.027^a^	0.067 ± 0.051^ab^	0.041 ± 0.052^bc^	0.043 ± 0.036^bc^	0.027 ± 0.011^c^	< 0.001	< 0.001
K02564: glucosamine-6-phosphate deaminase [EC:3.5.99.6]	0.018 ± 0.029^b^	0.056 ± 0.05^a^	0.061 ± 0.038^a^	0.053 ± 0.026^a^	0.072 ± 0.018^a^	< 0.001	< 0.001
K03092: RNA polymerase sigma-54 factor	0.095 ± 0.024^a^	0.065 ± 0.028^b^	0.044 ± 0.026^bc^	0.028 ± 0.015^c^	0.045 ± 0.014^bc^	< 0.001	< 0.001
K09860: outer membrane protein FlgP	0.087 ± 0.034^a^	0.032 ± 0.044^b^	0.014 ± 0.028^bc^	0.001 ± 0.004^c^	0.002 ± 0.003^c^	< 0.001	< 0.001
K10943: two component system, response regulator FlrC	0.000 ± 0.000^b^	0.000 ± 0.000^b^	0.000 ± 0.000^b^	0.000 ± 0.000^b^	0.002 ± 0.002^a^	< 0.001	0.001

^a, b, c^ mean values within a row with unlike superscript letters are significantly different (*q* < 0.05)

## Discussion

The present study aimed to investigate the microbial compositions and functional capacities of mucosal samples taken from the stomach, duodenum, jejunum, ileum, and mid-colon of dogs fed different diets. Although we did not observe differences in these dogs based on diets, our findings reveal important insights into the taxonomic composition and functional capacity of the microbiota along the canine GI tract. Consistent with previous research, we observed the greatest microbiota diversity in the mid-colon, aligning with the findings of Suchodolski et al. [6] who sampled intestinal contents from the duodenum, jejunum, ileum, and colon of healthy dogs, and Honneffer et al. [7] who collected intestinal content samples from the duodenum, ileum, colon, and rectum from adult healthy dogs. It is worth mentioning that the study by Suchodolski et al. [6] utilized 16S rRNA gene clone libraries analysis instead of Illumina high-throughput sequencing analysis, which was employed in our study. Additionally, both of these previous studies involved healthy adult hound dogs rather than beagles, which were the subjects of our study [6, 7]. Despite the differences in sample types, sequencing methods, dog breeds, and sampling sites, both investigations reported higher alpha-diversity measures in the colon and rectum. This concurrence is also supported by studies conducted in humans and rodents [25, 26].

The predominant phyla observed in all GI segments in present study were Proteobacteria, Firmicutes, Bacteroidetes, and Fusobacteria. These findings also align with previous studies that have analyzed both canine intestinal content samples [6, 7] and canine fecal samples [27–29]. Notably, we observed significant variations in the composition of the predominant bacterial phyla across GI tract segments. Specifically, Proteobacteria exhibited a decreasing trend from the stomach (80.8%) to the mid-colon (16.2%), which is in line with the findings reported by Honneffer et al. [7], where a gradual decline in Proteobacteria abundance from duodenum (59.1%) to rectum (4.8%) was reported. The relative abundance of Proteobacteria in the current study was predominantly attributed to the presence of *Helicobacter*, especially in the stomach, where *Helicobacter* accounted for approximately 95% of the Proteobacteria population. This finding is in line with previous studies in humans and pigs, where *Helicobacter* has been identified as a major constituent of the gastric microbiota [30, 31]. Within the mid-colon, *Anaerobiospirillum* was the major genus within the Proteobacteria phylum. *Anaerobiospirillum* is considered a normal part of microbiota of dog and cat feces, with several *Anaerobiospirillum* species being isolated from healthy and diarrheic dogs [32, 33].

Our study highlights Firmicutes as the predominant phylum in both the ileum (59.3%) and colon (40.1%) of the canine GI tract, with colon samples containing greater abundance of short-chain fatty acid producers, including *Blautia*, *Faecalibacterium*, and *Megamonas*. This observation aligns with the findings of Honneffer et al., where a greater abundance of Firmicutes was noted in the colon and rectum [7]. Furthermore, we observed that the mid-colon samples exhibited the highest relative abundance of Fusobacteria (19.5%), while the other regions showed a similar level of Fusobacteria, ranging from 2 to 4% of sequences. Our findings are similar to the results reported by Honneffer et al. [7], as they observed numerically greater Fusobacteria in the contents of the colon and rectum compared to the small intestine. This observation explains the relative abundance of Fusobacteria reported in studies primarily utilizing fecal samples, where abundances of Fusobacteria were typically found to be in the range of 20–30% [27, 28, 34]. Additionally, a dog study comparing microbiota in canine jejunal chyme and feces also observed greater counts of Fusobacteria in feces than in jejunal chyme, further supporting our results [35]. At the genus level, *Fusobacterium* exhibited the highest relative abundance (19.5%) in the mid-colon, followed by *Prevotella* (17.1%). This finding aligns with a previous dog study utilizing fecal samples, which identified *Fusobacterium* (25.4%), *Prevotella* (13.9%), and *Bacteroides* as the top three abundant bacterial genera in canine microbiota [36]. Despite variations in sample types and noted differences between mucosal and luminal microbiota [37, 38], these consistent patterns underline the stability of certain microbial compositions in specific GI segments across different studies, strengthening the reliability of our findings. The varying abundance of Fusobacteria and Firmicutes, particularly the pronounced increase in the mid-colon, offers intriguing prospects for further research to explore the roles, functions and its potential contribution to health or disease.

Bacteroides exhibited the highest abundance in the duodenum (25.6%) and remained consistent in the colon (17.1%) in our study. This aligns with previous findings by Honneffer et al. [7], who also noted no significant differences of Bacteroides abundance across GI segments of dogs, although there were numerical increases from duodenum (0.7%) to rectum (12.9%). Interestingly, a study involving human biopsy samples indicated a higher abundance of Bacteroides in the rectum compared to the duodenum [39]. The discrepancy in the results may be due to species differences, contributing to variations in Bacteroides distribution along the GI tract.

It is noteworthy that *Akkermansia*, a bacterial genus known to inhabit the mucus layer, was not identified in our study. Previous dog studies employing high-throughput Illumina sequencing have failed to detect *Akkermansia* in fecal samples [40–42]. This absence could be attributed to technical limitations such as sequencing depth and the inefficiency of 16S rRNA primers in capturing *Akkermansia*. Moreover, the divergence of *Akkermanisa*-like sequences from reference sequences may contribute to its absence in these studies [43].

The analysis of functional potential provides insights into the functional diversity of mucosal microbiota populations across various segments of the GI tract. Significant differences were observed in all L1 pathway categories among the GI tract segments, emphasizing the distinct functional differences of the microbiota in these segments. It is important to note that while significances were observed, certain pathway categories, such as those associated with human diseases, might not be directly applicable to the microbiota because PICRUSt was initially validated using data from humans.

The flagellar assembly pathway holds relevance within the bacterial realm, as flagella serve as an element for the motility of many bacteria. Consistent with findings form a previous dog study [7], our results showed notable differences in the abundance of several KOs linked to flagellar assembly (KEGG map02040) across GI segments. Notably, a majority of these altered KOs exhibited their highest abundance in the stomach. This observation indicates the presence of distinctive functional attributes among different GI segments.

Bile acid metabolism is one of the important roles partly performed by the gut microbiota [44, 45]. Within this process, the microbial enzyme, choloylglycine hydrolase, deconjugates bile salts. This enzyme has been identified across various bacterial taxa, including *Bacteroides*, *Clostridium*, *Lactobacillus*, and *Bifidobacterium* [46]. Honneffer et al. reported distinct presence of microbiota relevant KEGG pathway categories at L3, including primary bile acid biosynthesis (KEGG map00120), secondary bile acid synthesis (KEGG map00121) [7]. In the present study, the PICRUSt outcomes revealed a greater abundance of the KO to choloylglycine hydrolase (EC 3.5.1.24) in the mid-colon, which resonates with previous findings where this particular ortholog significantly increased from the duodenum to the rectum of dogs [7]. Moreover, the abundance of KO associated with secondary bile acid biosynthesis, especially 3-dehydro-bile acid delta 4,6-reductase (EC 1.3.1.114), was greater in the ileum and mid-colon. These observations are to be anticipated, as the primary site for both the deconjugation of bile salts and secondary bile acid synthesis is in the large intestine.

In this study, we investigated the potential impacts of diet and age on mucosal microbiota composition. Surprisingly, our results indicated that neither diet nor age significantly influenced the composition of the mucosal microbiota in the studied population. While both diet and age have been acknowledged as influential factors in shaping canine gut microbial communities in various contexts [8, 13, 14], the lack of substantial effects in this study may be attributed to several factors. First, the effect sizes for both diet and age were small, indicating that the study may be statistically underpowered, and consequently, differences between diets and ages might not be observed. Second, the dogs in our study were all housed in the same facility so they may have harbored relatively stable and resilient gut microbiota, potentially masking the potential influence of diet. A study in humans involving 159 individuals from 52 families revealed that household members shared a higher degree of similarity in their skin, oral and fecal microbiota compared to individuals from different households [47]. Another study in humans showed microbiota can revert to its baseline state over a 12-month period, following the initial shifts due to diet interventions [48]. It is plausible that the resilience of microbiota lead to a return to its original state after the dietary intervention and therefore, dietary effects were not observed in the current study. Additionally, despite differing in protein sources and a couple nutrient concentrations (e.g. fat; fiber), the diets were processed using the same methods (e.g., extrusion) and were of the same format (e.g., dry kibble). Nevertheless, these unexpected outcomes emphasize the complexity of gut microbiota interactions with diets and aging, indicating the need for further research encompassing diverse populations and dietary interventions.

In conclusion, this study contributes another rich database that supports our understanding of the mucosal microbiota populations along the GI tract of dogs. Our results were corroborated by previous research studies and highlight distinct compositional and functional attributes of the mucosal microbiota across different canine GI tract segments. While influences of diet or age on mucosal microbiota were not observed in this study, the bacterial taxonomic and predicted functional differences among GI segments provide valuable data that may stimulate further exploration of how the microbiota impact canine health and well-being.

## Electronic supplementary material

Below is the link to the electronic supplementary material.

Supplementary Material 1

## Data Availability

The sequence data generated from this study are available at the NCBI Sequence Read Archive (SRA; http://www.ncbi.nlm.nih.gov/sra) under accession number SUB14335230 and BioProject PRJNA1102005.

## Abbreviations

AAFCO: Association of American Feed Control Officials
GI: Gastrointestinal
KEGG: Kyoto Encyclopedia of Genes and Genomes
KO: KEGG Orthology
OTU: Operational taxonomic unit
PCoA: Principal coordinates analysis
PD: Phylogenetic diversity
PICRUSt: Phylogenetic Investigation of Communities by Reconstruction of Unobserved States
